# Amantadine therapy for Parkinson’s Disease: In Vivo Confocal Microscopy corneal findings, case report and revision of literature

**DOI:** 10.1186/s12886-022-02410-1

**Published:** 2022-05-10

**Authors:** Michela Cennamo, Francesco Dragotto, Eleonora Favuzza, Alberto Morelli, Rita Mencucci

**Affiliations:** Eye Clinic, Department of Neurosciences, Psychology, Pharmacology and Child Health, University of Florence, Largo Brambilla 3, 50134 Florence, Italy

**Keywords:** Amantadine toxicity, In Vivo Confocal Microscopy, AS-OCT, Endothelial Cell, Parkinson’s Disease, Case Report

## Abstract

**Background:**

To report a case of a patient showing bilateral corneal opacities after amantadine chronic treatment for Parkinson’s Disease (PD) and corneal edema associated with intra-epithelial and -endothelial depositions. After amantadine discontinuation a complete clinical remission with only a partial ultrastructural corneal recovery was reported.

**Case presentation:**

We describe a 78-year-old man with non-medical-responding bilateral corneal edema in treatment with systemic Amantadine for PD. In vivo confocal Microscopy (IVCM) analysis revealed hyperreflective particles at the epithelial level and expanded hyperreflective keratocyte and a disarrangement of stromal lamellae; endothelial cells showed hyperreflective intracellular inclusions in central and in peripheral areas with central polymegatism and pleomorphism. After 1 and 6 months the amantadine discontinuation, the absence of bilateral corneal edema and opacities were noted at the slit lamp examination, associated with the disappearance of epithelial and stromal abnormalities, but the persistence of endothelial hyperreflective deposits with a pleomorphism and polymegatism worsening at the IVCM exam.

**Conclusion:**

The evaluation of a patient’s cornea 6 months after the discontinuation of systemic amantadine therapy showed a clinical complete remission, with a complete resolution of the bilateral corneal oedema. On the other hand, ultrastructurally, amantadine toxicity is a completely reversible phenomenon at the epithelial level; conversely IVCM showed persistent endothelial degradation.

## Background

Amantadine, a systemic drug discovered in 1969 which acts as an antagonist of the N-MethylD-Aspartate-Type glutamate receptor, was primarily developed as a treatment for Type A viral influenza; since 1995 it has been widely used for the management of neurological disorders such as Parkinson’s Disease (PD) [1]. A large variety of amantadine-associated ocular complications have been described in literature, and in particular its toxic effect on endothelial cells with an increased risk of endothelial failure and a subsequent corneal oedema [2]. Several authors have hypothesized that the corneal oedema may be attributed to the presence of amantadine in the aqueous humor, with amantadine affecting corneal endothelial ion transport, which regulates corneal hydration [3].

However, amantadine can induce corneal alterations at each corneal layer. This can cause multiple-level corneal toxicity which acts as a cofactor in corneal oedema associated with normal Endothelial Cell Density (ECD) reduction [4]. We report, a case of a patient showing bilateral corneal opacities and corneal oedema associated with intra-epithelial and -endothelial deposits after amantadine treatment for PD, with complete clinical remission but only partial ultrastructural corneal recovery after the drug suspension. To the best of our knowledge, this case report describes for the first time ultrastructurally, by means of the In Vivo Confocal Microscopy IVCM, concomitant endothelial end epithelial abnormalities of an amantadine induced corneal oedema.

## Case presentation

A 78-year-old man was referred to the corneal service at the Ophthalmology Department at Careggi Teaching Hospital, Florence, Italy, in November 2020 for non-medical-responding corneal oedema. His medical history was composed of atrial fibrillation treated with anticoagulant therapy (Endoxaban) and PD treated with Levodopa and Amantadine (100 mg bis in die). The amantadine treatment had begun 2 years before. The patient reported progressively blurred vision in both eyes that had begun 3 months before the hospital visit and had been treated with bilateral topical and subconjunctival steroids. He denied a history of trauma, injuries, or surgery to either eye. His family history for corneal disease was negative.

**Fig. 1:**
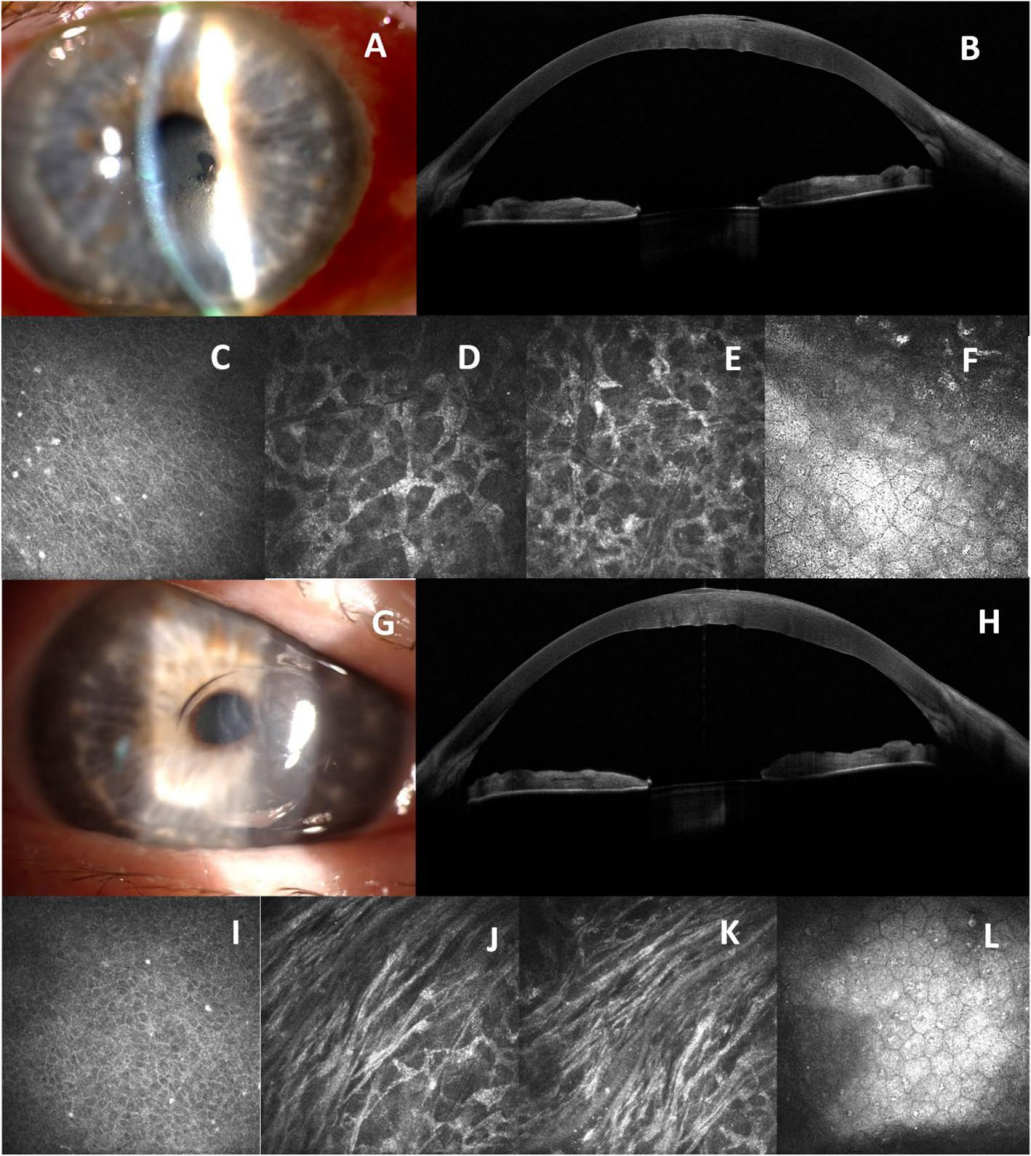
First consultation. **A** Slit-lamp examination of RE: central corneal oedema and vesicular opacities with Descemet plicae. **B** AS-OCT showed an increased CCT with para-central sub-epithelial bullae. **C**-**F** IVCM identified a few hyperreflective particles arranged with an irregular pattern at the epithelial level; expanded hyperreflective keratocyte cell bodies were observed in the stromal layer; CECs showed irregular hyperreflective intracellular inclusions. **G** Slit-lamp examination of LE showed central corneal oedema with Descemet folds confirmed by AS-OCT(**H**). **I**-**L** IVCM of LE revealed intra-epithelial hyperreflective deposits, as well as disarrangement of stromal lamellae due to central intrastromal oedema and endothelial inclusion with pleomorphism and polymegathism

At his first consultation his Best Corrected Visual Acuity (BCVA) was 20/60 Snellen equivalent in his Right Eye (RE) and 20/100 Snellen equivalent in his Left Eye (LE), Intraocular Pressure (IOP) was 14 mmHg in the RE and 15 mmHg in the LE. Slit-lamp examination revealed a central corneal oedema and vesicular opacities with Descemet plicae, little or no sign of anterior surface disorders, no signs of inflammation in the anterior chamber and no vitreous or retinal pathological findings (Fig. 1, A and G).

During the examination the following assessments were carried out in order to document corneal alterations: central ECD was 1106 cells/mm^2^ in RE and 1051 cells/mm^2^ in LE. The Corneal Endothelial Cells (CEC) located beyond these central areas were polymegathic (CEC enlargement) and pleomorphic (loss of CEC hexagonality), while the peripheral CECs showed only a slight degree of pleomorphism. The central pachymetry was 637 microns in RE and 683 in LE with a gradual thinning in the periphery.

The Anterior Segment Optical Coherence Tomography (AS-OCT, MS 39 CSO. Florence Italy) showed an increased central corneal thickness with sub-epithelial bullae in RE and a central corneal oedema with Descemet folds in LE (Fig. 1, B and H).

Several confocal scans were taken in the central and peripheral part of both corneas using IVCM (Heidelberg Retina Tomograph III Rostock Cornea Module HRT III-RCM Heidelberg Engineering GmbH, Germany). IVCM identified a few hyperreflective particles arranged with an irregular pattern at the epithelial level; there were no abnormalities in the Bowman layer, with a decrease in sub-basal nervous plexus density; in the stromal layer expanded hyperreflective keratocyte cell bodies and disarrangement of stromal lamellae were observed only in the central area, a sign of stromal oedema; CECs showed irregular hyperreflective intracellular inclusions both in the central and in the peripheral corneal area (Fig. 1, C-F, I-L).

An aqueous tap was performed to exclude the presence of a herpes virus in the LE; the PCR analysis was negative for herpes simplex viruses, varicella zoster virus and cytomegalovirus.

An adverse event related to a systemic drug reaction was suspected, amantadine has previously been reported as a cause of non-resolving corneal oedema. After a neurologist consultation the amantadine treatment was discontinued.

**Fig. 2:**
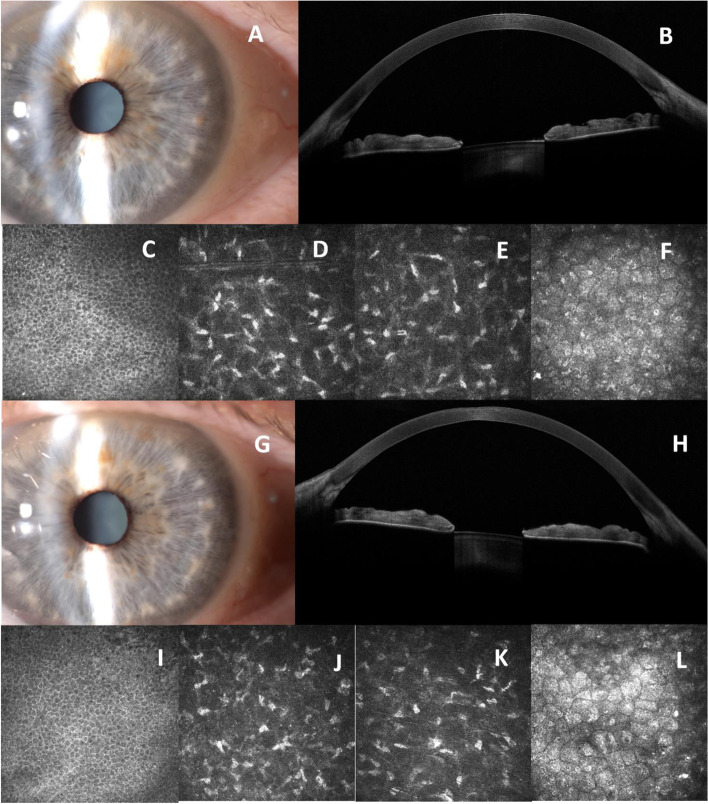
1^st^ month follow-up. **A** Slit-lamp examination of RE one month after discontinuing amantadine: absence of corneal oedema and opacities. **B** AS-OCT showed no corneal opacity or oedema in the RE. **C**-**F** IVCM revealed the disappearance of epithelial and stromal abnormalities with persistence of endothelial intracellular hyperreflective inclusions. **G** Slit-lamp examination of LE showed the disappearance of central corneal oedema. **H** AS-OCT revealed a slight corneal thinning with fine subepithelial opacity. **I**-**L** IVCM analysis of LE reported normal epithelial and stromal layers with a persistence of endothelial morpho-structural abnormalities: intracellular hyperreflective spots and a worsening of endothelial pleomorphism and polymegathism

One month later the patient was re-examined, and slit-lamp microscopy showed the absence of corneal oedema and opacities, bilaterally (Fig. 2 A, G). The patient’s BCVA returned to 20/25 Snellen equivalent in both eyes. AS-OCT showed no corneal opacity or oedema in the RE, Central Corneal Thickness (CCT) was 553 microns, a slight corneal thinning with a fine subepithelial opacity was reported in the LE, CCT was 568 microns, (Fig. 2 B, H). Corneal microstructural analysis revealed the disappearance of epithelial and stromal abnormalities and the persistence of endothelial intracellular hyperreflective inclusions, as well as a worsening of the endothelial pleomorphism and polymegathism, as confirmed by the analysis of the endothelial cell count (Fig. 2 C-F, I-L) (691 cell/ mm^2^ and 700 cell/ mm^2^ in the RE and in the LE, respectively). Six months after amantadine discontinuation the patient’s corneal opacities had disappeared, AS-OCT findings were normal and IVCM analysis showed a persistence of endothelial pleomorphism and polymegathism, with irregular intracellular hyperreflective deposits.

## Discussion and conclusion

CECs, a monolayer of hexagonal cells, are responsible for regulating stromal hydration and are metabolically active, preventing stromal overhydration and preserving the orderly arrangement of stromal collagen fibrils, which is necessary for corneal clarity. Mature CECs have limited regenerative capacity, and cell loss due to aging, disease, anterior segment surgery or drug toxicity results in stromal oedema and a loss of corneal transparency [5].

Amantadine, a pharmacological agent commonly used for PD management, affects the cornea in a cumulative, dose-dependent manner, as reported in previous studies. In particular, amantadine has shown an increased risk of endothelial pump failure or endothelial decompensation, characterised clinically as corneal oedema. A high cumulative use of amantadine generally causes corneal oedema within 2 years after the start of therapy [2, 6]. Amantadine-related corneal toxicity may concern not only the endothelium, but also involve corneal sub-basal nerve fiber layer density (SBNFLD), as highlighted in a prospective comparative observational study of 120 eyes. Patients with PD on amantadine therapy underwent a significant decrease in corneal SBNFLD in comparison with the amantadine naïve patients [7]. In our case report we found a reduction in the density of the SBNFL that could be related to the use of amantadine, or related to the patient’s underlying PD.

Furthermore, a recent case report showed highly reflective deposits in corneal epithelium, explained by two possible mechanisms: either a degenerative keratopathy phospholipidosis (PLD) in the cells of the corneal epithelium induced by amantadine, or a second mechanism related to a direct transport of amantadine into corneal epithelial cells through Organic Cationic Transporters [8].We found intra-epithelial bilateral hyperreflective deposits at the first examination, which were absent 1 month after discontinuing amantadine. Although the mechanism of intracellular accumulation is not yet clear, the epithelial deposits could be related to the drug in the tear film that is secreted from the lacrimal gland [9].

Even with a complete resolution of the corneal oedema, the endothelial abnormalities were still present even 6 months after suspending amantadine, with a progressive reduction of the ECD and with a worsening of the endothelial pleomorphism and polymegathism, as previously described by Chang et al. [9]. The degenerative process had been taken over rapidly, as shown by the rapid ECD loss from 1,106 to 691 cell/ mm^2^ in RE and from 1,051 to 700 cell/ mm^2^ in LE, month 1 to month 6, respectively. Moreover, 6 months after suspending amantadine the ultrastructural analysis performed using IVCM also showed a persistence of endothelial morpho-structural abnormalities and reported a persistence of endothelial intracellular hyperreflective spots.

However, similarly to what was described by Yoshinaka regarding corneal epithelial deposits, using the IVCM findings [8]. We were not able to ascertain whether the morphological findings of the highly reflective endothelial cells were due to PLD or amantadine deposits.

We hypothesize that intracellular degeneration phenomena persisted also after amantadine discontinuation, as the hyperreflective deposits persisted in number and size inside the endothelial cells. In our opinion, the use of amantadine causes decompensation and degeneration of endothelial cells, which mainly affects the central endothelium because its cellular aging process is characterized by a slow centripetal migration of less differentiated cells from the periphery to the center, throughout life. Older cells are more susceptible to decompensation, such as Fuchs Endothelial Corneal Dystrophy (FECD) pathogenesis [5]. Another possible hypothesis to explain why the endothelial damage is located in the central area could be the longer exposure of central cells to the amantadine which may be present in the aqueous humor, during systemic treatment. Finally, the clinical recovery could be justified by the functional compensation of the surviving endothelial cells which are no longer subjected to the amantadine pump failure effect, although this drug could trigger the process of apoptosis for some other cells, explaining the worsening of the endothelial pleomorphism and polymegathism.

The limits of this case report are the lack of knowledge of any preexisting altered baseline ECD and the endothelial parameters at the start of the amantadine therapy; in addition Levodopa can also be related to rare corneal toxicity as reported in a previous study [10].

IVCM is a useful diagnostic path, leading to a correct differential diagnosis of corneal deposition pathologies, and it excludes the presence of corneal dystrophy. It helped us to understand that despite the clinical resolution of the corneal oedema, endothelial ultrastructural anatomical alterations persisted.

In conclusion, amantadine toxicity is a completely reversible phenomenon at the epithelial level, thanks to the regenerative capacity of the epithelial tissue.

However, at the level of the endothelial cells, amantadine toxicity is reversible functionally, as shown by the disappearance of the corneal oedema, but it is anatomically irreversible, as detected by the persistence of intracellular deposits using corneal IVCM.

Further studies have to be conducted in order to confirm or discard our hypothesis. The authors think that an ophthalmological assessment should be conducted before amantadine treatment in PD patients.

## Data Availability

Data sharing is not applicable to this article as no datasets were generated or analyzed during the current study.

## Abbreviations

PD: Parkinson’s Disease
ECD: Normal Endothelial Cell Density
BCVA: Best Corrected Visual Acuity
RE: Right Eye
LE: Left Eye
IOP: Intraocular Pressure
CEC: Corneal Endothelial Cells
AS-OCT: Anterior Segment Optical Coherence Tomography
IVCM: In Vivo Confocal Microscopy
PLD: Phospholipidosis
FECD: Fuchs Endothelial Corneal Dystrophy
